# Street Food Handlers’ Knowledge and Hygiene Practices: A Descriptive Study

**DOI:** 10.7759/cureus.77894

**Published:** 2025-01-23

**Authors:** Basavant Dhudum, Yogesh Bhosale

**Affiliations:** 1 Department of Medical Surgical Nursing, Bharati Vidyapeeth (Deemed to Be University) College of Nursing, Sangli, IND

**Keywords:** food handlers, food hygiene, knowledge, practices, street food, street vendors

## Abstract

Introduction: Food safety is important to prevent foodborne illnesses from improper food handling by street food handlers. Food sold on the streets has become very common, especially in developing countries like India. Street foods are common in areas such as bus stops, schools, colleges, and street corners. Many of them are unhygienic and lack a basic understanding of the hygiene and handling of food. Knowledge and practices related to food sanitation and hygiene are important factors to consider in preventing foodborne illnesses.

Aim: This study aimed to assess the knowledge and practices related to food hygiene among street food handlers and develop a self-instruction module.

Methods: A descriptive study was conducted in selected areas of Sangli City, Maharashtra, India. The participants were 184 street food handlers working in selected areas in the Sangli district. This study was limited to vegetarian food handlers. Data were collected using a knowledge questionnaire and an observation checklist on practices regarding street food hygiene. The tool was validated by 17 experts, and reliability was tested using the Karl Pearson formula. The data were analyzed using frequency, percentage, and correlation coefficients.

Results: Out of the 184 participants in the study, 130 individuals (70.65%) demonstrated a moderate level of knowledge regarding food hygiene. Furthermore, approximately 146 participants (79.23%) adhered to food hygiene practices. However, despite the relatively high percentages of knowledge and practice observed, the relationship between the participant's knowledge of food hygiene and their actual practices was found to be weak. Statistical analysis revealed no significant correlation between these two variables, suggesting that possessing moderate knowledge about food hygiene does not necessarily translate into consistent practice of food hygiene measures among the participants.

Conclusion: Street food is a very common consumption of food among Indians. Food safety plays a vital role in the prevention of foodborne illness. The study findings showed that the majority of the participants have a moderate level of knowledge of food safety. Hence, there is a need to train street food handlers in food hygiene.

## Introduction

Access to safe and nutritious food is critical for survival and well-being. Street food is defined by the United Nations Food and Agricultural Organization (FAO) as ready-to-eat food and drinks that are made and/or sold by hawkers and vendors, particularly in streets and other such public areas [1].

According to the 2019 World Bank report on the economic burden of foodborne illnesses, the annual cost of treating foodborne illnesses is estimated to be $15 billion, and the total productivity loss linked to foodborne illness in low- and middle-income countries is estimated to be $95.2 billion. The number of individuals purchasing and consuming food made in public areas has increased due to urbanization and shifts in consumer behavior [2].

Due to its affordability and ease of access in cities, street food has become a significant part of people's diets and is ingrained in our contemporary way of life. However, a lot of street food vendors lack formal education and frequently are not aware of the fundamentals of food safety. Every day almost 2.5 billion individuals eat street food worldwide.

Although many street sellers have a rudimentary awareness of food safety concepts, professional training in sanitary procedures is frequently neglected [3]. While risks exist at every stage of the food supply chain, the majority of food safety issues arise during handling, preparation, storage, selling, and disposal. These critical stages are where food is most vulnerable to contamination, whether through improper hygiene, inadequate cooking temperatures, or unsanitary storage conditions. At the point of sale or consumption, mishandling can introduce pathogens or allergens, leading to foodborne illnesses. Effective food safety practices, including proper handling, cleaning, and temperature control, are essential to minimizing these risks and ensuring food safety for consumers, particularly in informal settings like street food stalls [4].

Inappropriate food storage (45%), cross-contamination (39%), and poor reheating or storage (50%) have all been linked to foodborne illnesses. Food contamination can be minimized by cleaning and disinfecting during food preparation because they remove potential germs. The chemicals that are used to clean or disinfect surfaces that come into touch with food must be safe [5].

In Southern Asia, as well as in countries such as Thailand, India, and Indonesia, street food plays a significant role in their diet. For women in developing regions, street-vended foods are also a crucial source of income, enabling self-employment and the chance to acquire business skills with minimal financial investment. A variety of ready-to-eat foods have been found in India. This typically includes quick Chinese meals, paranthas, puri bhaji, bhature, and kulcha, along with lighter snacks such as tea, biscuits, coffee, and cold drinks. This serves as a primary source of income for vendors, while consumers enjoy instant, delicious, and affordable meals. Consequently, street food vending in urban areas, particularly in metropolitan cities, has become an essential aspect of urban life and culture [6].

Food poisoning is the second most common cause of disease alerts and outbreaks in India over the past four years, with over 200 outbreaks reported by the National Centre for Disease Control under the Integrated Disease Surveillance Programme (IDSP) as of the 36th week of 2015 [7]. Focusing on these facts, this study highlights the knowledge and practices of street food handlers regarding food hygiene.

## Materials and methods

This study proposal was presented to the Institutional Ethics Committee of Bharati Vidyapeeth (Deemed to be University) College of Nursing, Sangli, and was approved to conduct the study (BVDU/CON/SAN/16/2023-2024). Permission to conduct the study was obtained from the concerned authority of the city municipal corporation.

We conducted a cross-sectional descriptive study to assess the knowledge and practices regarding food hygiene among street food handlers in selected areas of Sangli City, Maharashtra, India.

The objectives of the study were, first, to assess the level of knowledge and practices related to food hygiene among street food handlers. Second, to correlate knowledge and practices related to food hygiene; third, to determine the association between knowledge and demographic variables; and finally, to develop a self-instructional module on food hygiene for street food handlers.

We calculated the required sample size based on prevalence, and a total of 184 samples were selected using the purposive sampling technique. This study included food handlers who sold only vegetarian food, held licenses from municipal corporations, and were aged between 18 and 70. The food handlers who sold non-vegetarian food and who did not give consent were excluded from the study. Participants were given all the necessary information related to the study, and written informed consent was obtained from the participants before data collection.

We collected the data using a knowledge questionnaire and observation checklist related to food hygiene from 26th April 2024 to 30th April 2024. Seventeen experts from the nursing field validated the tool, and the reliability of the knowledge questionnaire was measured by the split-half technique and Karl Pearson’s formula. We tested the observation checklist by the inter-rater method. The r value of the knowledge questionnaire was 0.84 and that of the observation checklist was 0.81. Hence, the tool was found to be reliable for this study. The authors constructed the structured knowledge questionnaire on food hygiene. There were 12 multiple-choice questions related to food hygiene. 

Each question was given four options, and the right answer was given one mark. The participants have to tick mark the right option. A knowledge score between one and four was considered a poor level of knowledge, between five and eight was considered a moderate level of knowledge, and between nine and 12 was considered a good level of knowledge. A knowledge questionnaire related to food hygiene is given in Table 1.

**Table 1:** Knowledge questionnaire related to food hygiene

S.No.	Knowledge questionnaire
1	What is the most important reason for washing hands before handling food?
2	Which of the following is a key practice to prevent cross-contamination?
3	What temperature should hot food be kept at to ensure it is safe?
4	How often should food contact surfaces be cleaned and sanitized?
5	Which of the following is a common symptom of foodborne illness?
6	What is the best way to prevent foodborne illness caused by bacteria?
7	Which of the following is a common food allergen that must be identified to the customers?
8	When used excessively in food, which of the following chemicals can lead to hormonal disruption?
9	Which chemical additive, commonly used as a preservative, has been linked to an increased risk of cancer?
10	Which of the following is the best practice for storing perishable food items to prevent spoilage?
11	Which chemical is often used to artificially ripen fruits, posing health risks?
12	Which of the following is a common adulterant found in chili powder?

The structured observation checklist is designed to assess the food hygiene practices of street food handlers. It contains a list of specific actions or practices related to food safety, such as proper handwashing, use of gloves, cleanliness of utensils, food storage, and handling procedures. Each practice is listed with two columns next to it: one for marking if the practice is "followed" and the other for marking if it is "not followed." An observation checklist related to food hygiene is given in Table 2.

**Table 2:** Observation checklist related to food hygiene

S.No.	Observation statements on food hygiene
1	Washes hands before and after handling food
2	Wears clean cloth
3	Wears clean cap
4	Wears a clean apron
5	Uses hand gloves during cooking and handling food or serving
6	Keeps separate and covered dustbins for discarding food waste
7	Uses clean water for food preparation
8	Stores food in separate containers
9	Uses clean and separate dusters to clean surfaces and wipe utensils
10	Washes utensils in the clean water

Statistical analysis

First, we tabulated the collected raw data on a Microsoft Excel (Microsoft® Corp., Redmond, WA) sheet and organized it into demographic variables and knowledge scores. The data was summarized in frequency and percentage, and the association between knowledge scores and demographic variables was assessed by using the chi-square test. We determined the correlation between knowledge and practices by using Pearson's correlation coefficient. The acceptance level of significance was P-value <0.05. The data was presented in tables and graphs.

## Results

The authors organized the data in various sections. First, we assessed the demographic variables and presented them as frequency and percentage. Second, we assessed knowledge and practices and presented them as frequency and percentage. Third, we identified the correlation between knowledge and practices, and finally, we determined the association between knowledge score and demographic variables.

The ages of the participants ranged between 18 and 50 years, and most of the participants ranged between 25 and 50 years (59.78%). There were 133 (72.28%) male participants and 51 (27.72%) female participants. A total of 80 (43.48%) participants had completed primary education, and 77 (41.85%) had completed secondary education. About 136 (73.91%) of the participants ran a food vending business for over 10 years. A maximum number of 138 (75%) participants had undergone food hygiene training. The results of the demographic variables are summarized in Table 3.

**Table 3:** Frequency and percentage distribution according to the demographic variables

S. NO.	Demographic variables	Frequency	Percentage
1.	Age in years		
	18-25	54	29.35%
	25-50	110	59.78%
	50-70	20	10.87%
2.	Gender		
	Male	133	72.28%
	Female	51	27.72%
3.	Educational status		
	No formal education	23	12.50%
	Primary education	80	43.48%
	Secondary education	77	41.85%
	Graduation and above	4	2.71%
4.	Duration of food vending (years)		
	>10	136	73.91%
	10-15	32	17.39%
	<15	16	8.70%
5.	Training on food hygiene		
	Yes	138	75.00%
	No	46	25.00%

**Figure 1:**
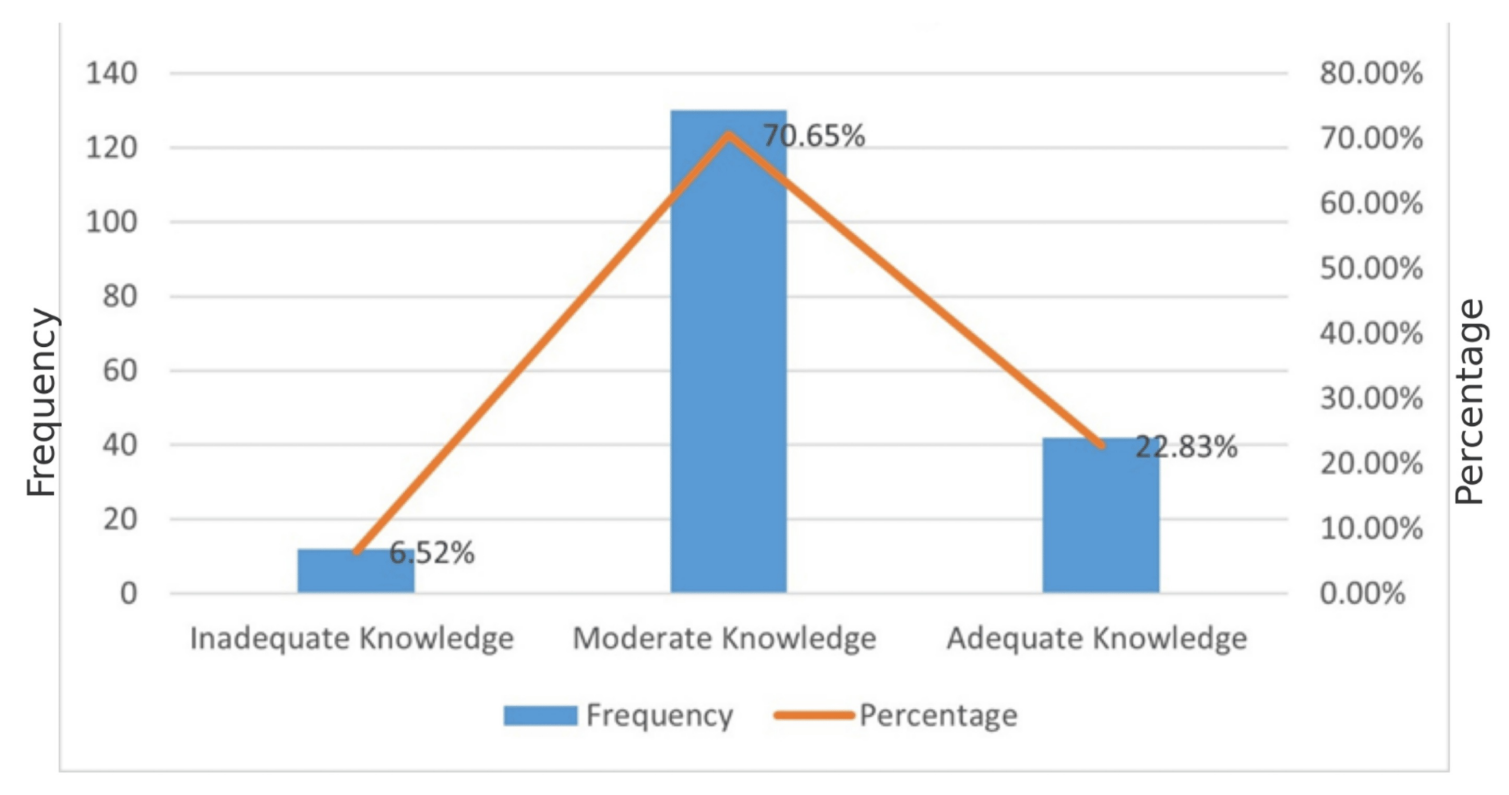
Level of knowledge regarding food hygiene among street food handlers

A total of 130 (70.65%) participants had a moderate level of knowledge, 42 (22.83%) of them had adequate knowledge, and only 12 (6.25%) had poor knowledge of food hygiene. The findings of the knowledge level are presented in Figure 1.

About 79.23% (mean percentage) of the participants followed almost all the practices related to food hygiene, and only 20.77% of them followed only a few hygienic practices. The findings of the practices related to food hygiene among street food handlers are presented in Table 4.

**Table 4:** Practices related to food hygiene among food handlers

Practice items regarding food hygiene	Followed frequency	Followed percentage	Not followed frequency	Not followed percentage
Washes hands before and after handling food	184	100%	Nil	Nil
Wears clean cloth	144	78.26%	40	21.74%
Wears clean cap	156	84.78%	28	15.22%
Wears the apron	78	42.40%	106	57.60%
Uses hand gloves during cooking and handling food	121	65.76%	63	34.24%
Keeps separate and covered dustbins for food waste	154	83.69%	30	16.31%
Uses clean water for food preparation	174	94.56%	10	5.44%
Stores food in separate containers	168	91.31%	16	8.69%
Uses clean and separate dusters to clean surfaces	95	51.63%	89	48.37%
Washes utensils in the clean water	184	100%	Nil	Nil
Total	1458		382	
Average	145.8	79.23%	38.2	20.77%

The mean for knowledge is 7.03 with an SD of 2.41, while practices have a mean of 7.8 and an SD of 1.19, indicating slightly more variability in knowledge. The Pearson correlation coefficient (r=0.03789) suggests a weak positive linear relationship between the two variables. The coefficient of determination (r2=0.001436) shows that only 0.1436% of the variance in practices is explained by Knowledge. The P-value (0.6096) indicates this relationship is not statistically significant, with a sample size of 184. The findings of the correlation between knowledge and practices are summarized in Table 5.

**Table 5:** Correlation between knowledge and practices related to food hygiene

Parameter	Value
Mean (knowledge)	7.03
SD (knowledge)	2.41
Mean (practices)	7.8
SD (practices)	1.19
Pearson correlation coefficient (r)	0.03789
Coefficient of determination (r²)	0.001436
P-value	0.6096
Covariance	0.1102
Sample size (n)	184

The study showed a significant association between knowledge level and educational status and knowledge level and training in food hygiene (p-value <0.05). There is no significant association between knowledge scores and age, gender, or duration of food vending (p-value >0.05). The details are shown in Table 6.

**Table 6:** Association between knowledge level and demographic variables

Demographic variable	Chi-square	P-value	Significant association
Age	1.32	0.858	No
Gender	2.16	0.339	No
Educational status	15.01	0.020	Yes
Duration of food vending	4.28	0.370	No
Training on food hygiene	23.65	0.000007	Yes

## Discussion

The study evaluated the knowledge and practices of food handlers regarding food hygiene. Most food handlers (70.65%) had moderate knowledge, 22.83% had adequate knowledge, and 6.52% had inadequate knowledge.

In terms of hygiene practices, all food handlers washed their hands before and after handling food and washed utensils in clean water. A majority wore clean clothes (78.26%), clean caps (84.78%), and used clean water for food preparation (94.56%). However, only 42.40% wore aprons, and 65.76% used hand gloves during cooking and handling food. Additionally, 83.69% kept separate and covered dustbins for food waste, 91.31% stored food in separate containers, and 51.63% used clean and separate dusters to clean surfaces.

The Pearson correlation coefficient showed a non-significant, very small positive relationship between knowledge scores and practice scores (r(182)=0.0379, p=0.610). Educational status and training on food hygiene were significantly associated with knowledge scores, highlighting the importance of education and training in improving food hygiene knowledge and practices.

These findings are consistent with the study by Ateye et al., which reported that most participants (67.50%) had adequate knowledge of food hygiene. This similarity highlights the general awareness of food hygiene among the populations studied. While our study showed a higher percentage of participants with moderate knowledge, the results suggest that knowledge levels vary but are generally sufficient to promote safe practices. However, despite adequate knowledge in some cases, translating this into consistent practices remains a challenge, as seen in the weak correlation between knowledge and practices in our study [8].

A study by Francis et al. (2024) in Nigeria found that 67% of food vendors had good knowledge of food hygiene, 70% displayed positive attitudes, but only 53% practiced food hygiene correctly. This highlights a gap between knowledge, attitudes, and actual practices, similar to our study's findings. While awareness and attitudes toward food hygiene may be high, ensuring that this translates into proper practices remains a significant challenge [9].

Mukherjee S et al. (2018) reported that 88.7% of participants were aware of food hygiene and recognized the link between poor food hygiene practices and foodborne illnesses. This highlights the critical role of knowledge in preventing foodborne diseases. The findings suggest that awareness campaigns and educational interventions are effective in increasing knowledge about food safety. However, knowing alone may not be sufficient to ensure proper practices, as seen in our study, where knowledge did not strongly correlate with practice. This underlines the need for targeted efforts to translate awareness into consistent food hygiene practices to reduce the risk of illnesses [10].

Choudhury M et al. concluded that training street food vendors on food hygiene practices is essential. The study emphasized that many vendors lack the proper knowledge and skills to handle food safely, increasing the risk of foodborne illnesses. Providing structured training programs can help vendors understand the importance of maintaining hygiene during food preparation, storage, and handling. Such initiatives can improve their practices, ensuring safer food for consumers. The findings align with our study, which highlights the gap between knowledge and practice, stressing the need for continuous education and monitoring to promote better hygiene standards among food handlers [11].

However, other studies show that good knowledge does not always translate into safe food handling practices, so even though knowledge and practice levels seem adequate, the gap between knowledge and actual practice is still a concern. According to Saurabh R. K et al., 2015 study results showed the majority of the street food vendors, 82.5%, were not certified in food training, whereas we found the majority (75%) of the street food handlers had undergone training on food hygiene. The authors also stated the majority of the participants followed all the practices related to food hygiene [12].

A study by Sufia Islam et al. in Bangladesh found that higher education and training are strongly linked to better knowledge and practices of food hygiene. This highlights the importance of providing regular training programs and educational initiatives to improve food hygiene standards. Educating individuals, especially food handlers, can help them understand the importance of proper hygiene during food preparation and handling. These targeted interventions can bridge the gap between knowledge and practice, ensuring safer food practices. The findings align with our study, emphasizing the need for continuous efforts to enhance food hygiene awareness and implementation [13].

The study by Abid MT et al. (2022) assessed the food safety knowledge, attitudes, and practices of street food vendors in Chattogram City, Bangladesh. The findings highlighted significant gaps in the vendors' understanding and implementation of food safety measures, despite many demonstrating positive attitudes toward hygiene. The study emphasized the urgent need for targeted training programs and stricter regulations to improve food safety practices. These efforts could minimize health risks and enhance public safety. Adding this to our findings further underscores the importance of education, regular training, and monitoring to bridge the gap between knowledge and practice in food hygiene [14].

In conclusion, enhancing food hygiene practices requires a combination of education, regular training, and consistent monitoring. Studies highlight that while knowledge and attitudes toward food hygiene are often adequate, there is a gap in translating this into proper practices. Training programs tailored to food handlers and street vendors can bridge this gap by equipping them with the necessary skills and awareness. Additionally, continuous monitoring and follow-up are crucial to ensure compliance and reinforce good practices. By implementing these measures, the risk of foodborne illnesses can be significantly reduced, leading to improved public health and safer food for consumers.

The limitation of this study is that it was conducted among selected areas of Sangli city and only among vegetarian street food handlers, with a small sample size of 184. Hence the findings of the study cannot be generalized to a larger group. Therefore, the authors suggest conducting studies on a larger sample size and including both vegetarian and non-vegetarian food handlers.

## Conclusions

The study findings conclude that the majority of street food handlers have a moderate level of knowledge and follow almost all food hygiene practices. We found an association between knowledge level, educational status, and training in food hygiene. Street foods are among the most common, easily available, and affordable foods on urban streets. They also serve as one of the important sources of income. However, these street foods do not adhere to hygienic standards; they can cause food-borne illnesses that can cause morbidity and mortality as well as have an impact on the development of the country. Therefore, educating and training street food handlers is crucial, as many do not adhere to all hygienic practices.
